# The state of the art of bispecific antibodies for treating human malignancies

**DOI:** 10.15252/emmm.202114291

**Published:** 2021-08-24

**Authors:** Shuhang Wang, Kun Chen, Qi Lei, Peiwen Ma, Andy Qingan Yuan, Yong Zhao, Youwei Jiang, Hong Fang, Shujun Xing, Yuan Fang, Ning Jiang, Huilei Miao, Minghui Zhang, Shujun Sun, Zicheng Yu, Wei Tao, Qi Zhu, Yingjie Nie, Ning Li

**Affiliations:** ^1^ Clinical Cancer Center/National Cancer Center/National Clinical Research Center for Cancer/Cancer Hospital Chinese Academy of Medical Sciences and Peking Union Medical College Beijing China; ^2^ NHC Key Laboratory of Pulmonary Immunological Diseases is supported by the non‐profit Central Research Institute fund of Chinese Academy of Medical Sciences (2019PT320003) Guizhou Provincial People’s Hospital Guiyang China; ^3^ Excyte Biopharma Ltd Beijing China; ^4^ Excyte LLC Rockville MD USA; ^5^ Nanjing Umab‐biopharma Co., Ltd Nanjing China; ^6^ Hangzhou Genekine Biotech Co., Ltd Hangzhou China; ^7^ Department of Medical Oncology Harbin Medical University Cancer Hospital Harbin China; ^8^ Queen Mary School Nanchang University Nanchang China; ^9^ Geneplus Shenzhen China; ^10^ China Pharmaceutical University Nanjing China

**Keywords:** bispecific antibodies (bsAb), malignancies, single‐chain variable fragment (scFvs), tumor associate antigen, tumor‐specific antigen, Cancer, Immunology

## Abstract

Bispecific antibodies (bsAb) that target two independent epitopes or antigens have been extensively explored in translational and clinical studies since they were first developed in the 1960s. Many bsAbs are being tested in clinical trials for treating a variety of diseases, mostly cancer. Here, we provide an overview of various types of bsAbs in clinical studies and discuss their targets, safety profiles, and efficacy. We also highlight the current challenges, potential solutions, and future directions of bsAb development for cancer treatment.

## Graphical abstract

In this review, N. Li, Y. Nie & colleagues provide an overview of the current clinical development and future directions of bi‐specific antibodies for cancer treatment.

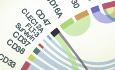

**Glossary**
Monoclonal antibody (mAb): A mAb is made by cloning individual white blood cells and is specific for only one antigen or epitope. Monoclonal antibodies are widely used in cancer therapy to block cell growth, flag cancer cells for destruction, or trigger other mechanisms to kill cancer cells.
Bispecific antibody (bsAb): A bsAb is designed to bind two different targets or epitopes and can thereby exert two different functions. They are currently used to treat infectious, inflammatory, and malignant diseases.
Fragment crystallizable region (Fc): The Fc is the tail region of the immunoglobulin molecule, which contains only the constant region of the heavy chain and binds to effector molecules.
T‐cell receptor (TCR): TCR is a T‐cell surface complex responsible for recognizing antigens and is stimulated by major histocompatibility complex molecules.
T‐cell‐redirecting bispecific antibody (TRBA): TRBA is a bivalent antibody that binds to CD3 on T cells and a cancer cell antigen in order to recruit T cells to kill cancer cells.
Bispecific T‐cell engagers (BiTE): BiTE are a subtype of bispecific antibodies, which are constructed by connecting two single‐chain variable fragments via a flexible linker. One fragment binds to a tumor‐associated antigen, and the other binds to a T‐cell‐specific antigen to activate the T cell to kill the cancer cell to which it is linked.
Dual‐affinity re‐targeting antibody (DART): DART consists of two variable fragments connecting the opposite heavy chain variable regions by a sulfide bond, which improves the stability.
Tumor associate antigen (TAA): TAAs are antigens mainly arising from genetic amplification or post‐translational modification that are expressed on tumor cells and a subset of normal cells. TAAs are usually expressed preferentially higher in tumor cells.
Tumor‐specific antigen (TSA): TSAs are antigens mainly arising from oncogenic driver mutations that generate novel peptide sequences. TSAs are only expressed on tumor cells and not present in normal cells.
Cytokine release syndrome (CRS): CRS is a systemic inflammatory response triggered by infections, chemical drugs, or biological therapies. CRS is a serious adverse effect of T‐cell‐engaging immunotherapies such as bispecific antibodies and chimeric antigen receptor T‐cell therapies.

## Definition and classification

A bispecific antibody (bsAb) is designed and manufactured—through genetic recombination, chemical conjugation or quadromas—to contain two target‐binding units in one antibody‐based molecule, whereby each unit independently recognizes its unique epitope. Upon sequential or simultaneous binding, bsAb acts as a biophysical bridge between two antigens with multiple mode of actions (MoA) *in vivo* to achieve specific effects.

Basic research and development have yielded many different forms of bsAb with different properties. Their classification can be based on various criteria, for example, the length of their half‐life *in vivo*. Here, bsAbs can be roughly divided into two groups. The first one are small proteins, usually less than 50 kDa, generated by fusion of two basic single‐chain variable fragment (scFvs) or two single‐domain units. Termed bispecific T‐cell engager (BiTEs; Löffler *et al*, 2000), dual‐affinity re‐targeting antibody (DARTs; Johnson *et al*, 2010), or diabodies (Holliger *et al*, 1996), they lack a human immunoglobulin constant region (Fc) which leads to quick clearance *in vivo* within a few hours. Accordingly, these smaller bsAbs have no Fc‐mediated effector functions and require continuous administration for therapeutic use. The other group are long‐lived bsAbs (> 150 KDa) with a half‐life of up to several days *in vivo*. These include bsAbs with a human Fc and a classic antibody backbone similar to traditional IgG (Ridgway *et al*, 1996), and recent scFv‐IgG fusion bispecific antibodies (Shen *et al*, 2006) or similar assemblies.

The US FDA grouped bsAbs into two main classes based on their mechanism of action, namely cell‐bridging bsAbs and antigen‐crosslinking bsAbs (non‐cell‐bridging molecules; Labrijn *et al*, 2019). Most cell‐bridging bsAbs are designed for cancer treatment by linking immune cells to malignant cells. Through sequential binding, that is, by binding the cancer cell first owing to a higher affinity to tumor antigens, cell‐bridging bsAbs can improve specificity and effectiveness with reduced non‐specific side effects and lower dosage compared with mAbs. In contrast, antigen‐crosslinking bsAbs target two antigens or two receptors simultaneously. Their main MoA is either blocking signals of cell growth/survival or activation of immune cells (Engelman *et al*, 2007). Antigen‐crosslinking bsAbs basically act similar to mAbs except that they bind two different targets.

**Figure 1:**
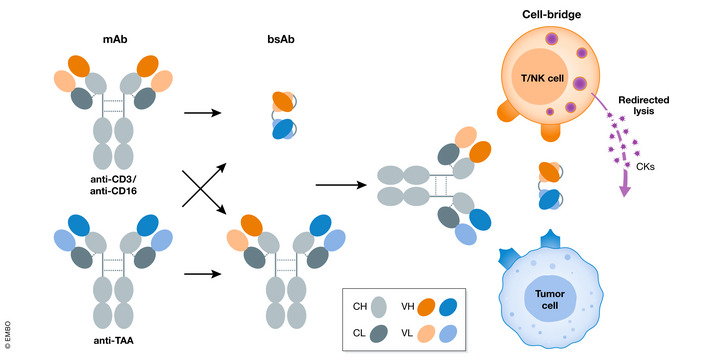
Schematic diagram of cell‐bridging bispecific antibodies The basic bsAb construct is designed as two connected units, one specific to T cells (CD3) or NK cells (CD16) and the other to a tumor‐associated antigen (TAA). In terms of *in vivo* half‐life, bsAbs can be divided into two groups. Group one includes bsAbs small in size, typically consisting of two basic single‐chain fragments with variable domain. The other group includes large and long‐lasting bsAb with a full antibody backbone, structurally akin to classic IgG. (CH: heavy chain constant region, CL: light chain constant region, VH: heavy chain variable region, VL: light chain variable region, CK: cytokines).

bsAbs have been used clinically in regenerative medicine and to treat infectious diseases such as HIV (Huang *et al*, 2016), hematological disorders, and cancer depending on their design and MoA. More than 85% of bsAbs in clinical trials are cancer therapeutics, of which more than 50% are cell‐bridging bsAbs in small or large assembly formats (Fig 1). The basic anti‐cancer bsAb construct usually recognizes a tumor‐associated antigen (TAA) and either T cells usually via CD3 (Clark & Waldmann, 1987) or NK cells usually via CD16 (Oberg *et al*, 2018; Thakur *et al*, 2018).

## Overview of worldwide cancer‐related bsAb clinical trials in the past 10 years

**Figure 2:**
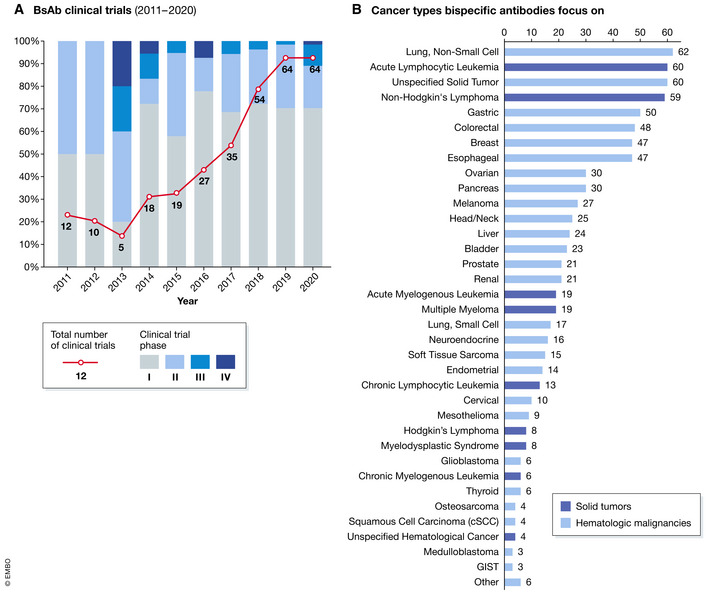
(A) History and phase distribution of bsAb clinical trials worldwide during the past ten years (2011‐2020); (B) cancer types that bispecific antibodies are being used against Details of the trials were obtained from Pharmaprojects, a drug development database developed by INFORMA (https://pharma.id.informa.com). The following search keywords were used as follows: [(Therapeutic Class is Antiboby, bispecific, T cell engager) OR (Therapeutic Class is Antibody, bispecific)] AND (Actual Start Date is from 2010/01/01 to 2020/08/01)].

Although the concept of bsAb was first proposed in 1964 (Nisonoff *et al*, 1960), its translation into clinical practice took several decades and had to address multiple problems in basic research and manufacturing. It was not until 2014 when the first product was approved: Blincyto, an anti‐CD19 X anti‐CD3 bispecific antibody (BiTE), gained FDA approval for treatment of relapsed or refractory B‐cell acute lymphoblastic leukemia (Przepiorka *et al*, 2015). Encouraged by the success of Blincyto, a considerable number of new clinical trials have been registered since 2014. Moreover, the number of new clinical trials evaluating novel bsAb drugs has been continuously increasing with an annual rate of 20.44% (Fig 2A). Of particular importance, a similar number of trials targeting solid tumors were observed compared with those targeting hematological malignancies (Fig 2B, solid tumors 170/308, 55.2% vs hematological tumors 138/308, 44.8%). Nonetheless, more than 93.5% of the trials are still in phase I or II.

Here, we discuss the clinical characteristics and related details of bsAbs in clinical trials for cancer treatment between January 1, 2010, and August 1, 2020, based on data from Trialtrove and Pharmaprojects, a drug development database developed by INFORMA. In total, 308 projects investigating 126 bsAb drugs were analyzed.

### bsAbs for treating hematological tumors

**Figure 3:**
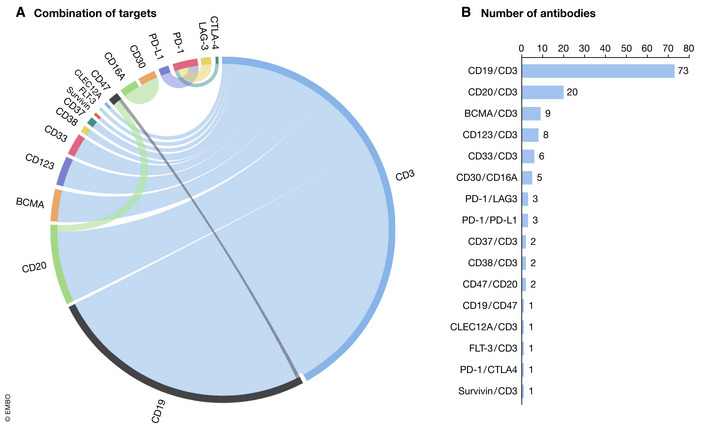
(A) Target combinations of bispecific antibodies in hematologic malignancies (combination of targets); (B) target combinations of bispecific antibodies in hematologic malignancies (number of antibodies) Details of the trials were obtained from Pharmaprojects, a drug development database developed by INFORMA (https://pharma.id.informa.com). The following search keywords were used as follows: [(Therapeutic Class is Antiboby, bispecific, T cell engager) OR (Therapeutic Class is Antibody, bispecific)] AND (Disease is Oncology: Unspecified Hematological Tumor) AND (Actual Start Date is from 2010/01/01 to 2020/08/01)].

The 138 bsAb programs treating hematological tumors focused on 16 different targets, including CD19, CD20, BCMA, CD123, and CD33 (Fig 3A and B). Among them, more than half (73/138) were CD19‐targeting cell‐bridging bsAbs, probably trying to replicate the success of Blincyto. The second largest group targeting CD20, BCMA, and CD123 accounted for about one quarter of hematological cancer trials. The lion’s share of bsAbs in these trials (129/133) redirected T cell (CD3) to cancer cells. Only five bsAb programs bridge NK cells by targeting CD30 and CD16. Antigen‐crosslinking bsAbs accounted for just a tiny fraction (4/138).

### Landscape of bsAb in treating solid tumors

**Figure 4:**
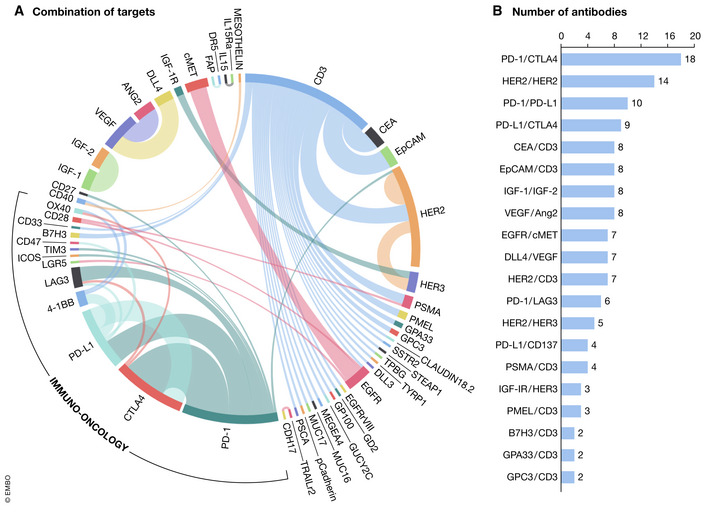
(A) Target combinations of bispecific antibodies in solid tumors (combination of targets); (B) target combinations of bispecific antibodies in solid tumors (number of antibodies) Details of the trials were obtained from Pharmaprojects, a drug development database developed by INFORMA (https://pharma.id.informa.com). The following search keywords were used: [(Therapeutic Class is Antiboby, bispecific, T cell engager) OR (Therapeutic Class is Antibody, bispecific)] AND (Disease is Oncology: Unspecified Solid Tumor) AND (Actual Start Date is from 2010/01/01 to 2020/08/01)].

Solid tumors account for 90% of newly diagnosed cancer cases, but very few drugs are available to produce durable therapeutic benefits in patients, owing to the high heterogeneity of cancer cells, relatively low level of neoantigens on cancer cells, and the inhibitory tumor microenvironment. Notwithstanding, there are currently more than 170 clinical trials of bsAbs to treat solid tumors, targeting 56 molecules and/or their rational pairs (Fig 4A).

Compared to hematological cancer trials, bsAbs for treating solid tumors involve many more targets and multiple MoAs. While both immune cell‐redirecting bsAbs and antigen‐crosslinking bsAbs are being explored, the latter account for more than three fourth on these studies (99/135; Fig 4B). The crosslinked antigen pairs vary from single targets such as HER2/HER2 (biparatopic bsAb) to combinations of two different proteins, for example, VEGF/Ang‐2, IGF‐1/IGF‐2, PD‐1/CTLA4, PD‐1/PD‐L1, or PD‐L1/CTLA4. Of note, many bsAbs (47/135 in Fig 4B) are being tested to target immune cells with the expectation that they outperform single mAbs (such as anti‐PD‐1 mAb) in modulating anti‐cancer functions. The MoA of these bsAbs is mainly by targeting dual cell signaling pathways for enhanced inhibitory or stimulatory effects in malignant cells or immune cells accordingly. Immune cell‐redirecting, anti‐CD3/anti‐TAA‐based cell‐bridging bsAbs on the other hand utilize not only tissue‐specific tumor antigens (PSMA, GPC3, etc.) but also fewer specific antigens (HER2, EpCAM, CEA, etc.).

Though some of the targets (HER2, EGFR, etc.) have been validated clinically, no bsAb drug against solid tumors has been approved to date. Nevertheless, a subgroup of cell‐bridging bsAbs with CD3 as the constant target in T cells, termed TRBAs (T‐cell‐redirecting bispecific antibody), have begun to dominate novel cancer therapies from scientific research to clinical studies.

## Modes of actions of bsAb

bsAbs are designed to achieve different functions through single or multiple MoAs: bridging tumor cells and immune cells for redirected cytotoxicity, blocking two targets to inhibit tumor growth, promoting immune cell functions, or facilitating the formation of protein complexes (Hemlibra, which is not the scope of this review).

### Bridging tumor cell and immune cells

Cellular immune surveillance usually recognizes and eliminates abnormal cells by bridging them with a cytotoxic T cell via the TCR‐MHC‐peptide complex. Malignant cells may escape this link by lowering expression of immunological HLA peptide, in addition to secreting immune suppression molecules and creating a hostile tumor microenvironment for infiltrating T cells. The main aim of CD3‐ or CD16‐targeting, TAA‐based bsAbs is to create new and stable bridges between immune cells and tumor cells and thereby enhance the efficacy and coverage of T cell and NK cell action.

Modulating the bridging strength may be key to optimizing clinical outcome. Blincyto, MGD011, and AFM11 were all designed to link T and B cells through CD3‐CD19 bsAbs and had different cytokine release syndrome (CRS) profiles in clinical trials. Glofitamab, epcoritamab, and mosunetuzumab all are CD20×CD3 redirecting T cells to CD20‐positive cells for treating B‐cell malignancies by manipulating the bridging strength, in addition to modulating the affinities of the two moieties in a bsAb. Glofitamab was designed as a 2:1 antibody with two CD20 binding points and one CD3 binding point to achieve higher affinity against malignant cells and relatively lower affinity to immune cells to prevent massive immune activation and damage to normal tissues and cells. Epcoritamab designed with the DuoBody platform was subcutaneously administered with a better safety profile. In early‐phase trials, there was no grade 3 CRS observed in patients treated with epcoritamab. Mosunetuzumab was a fully humanized IgG1‐like bispecific antibody, investigated in R/R NHL and DLBCL, that produced a significant number of complete response among patients with acceptable safety profiles. NK cell can also be the effector in this type of bsAbs, by adopting the CD16 antibody, such as in AFM13 or GTB‐3550.

### Blocking two targets

Past conventional mAb therapeutics and small molecule drugs in oncology have unveiled one of the dominant resistance mechanisms by cancer cells: using alternative growth signals to compensate the blockade. Blocking two signal targets simultaneously, besides other antibody‐mediated cytotoxicities, may overcome such resistance and enhance efficacy and coverage. This, however, requires careful design for bsAbs to balance the two antibody affinities to two targets as well as the spatial distance for sufficient and concurrent bivalent interaction.

Amivantamab is such a bsAb that targets cMet and EGFR. It has been granted approval to treat non‐small‐cell lung cancer patients with resistant malignancies after several lines of tyrosine kinase inhibitors and patients with EGFRex20mut who lack other effective treatment. Amivantamab was investigated in patient subgroups with overall response rate (ORRs) greater than 30%. It effects multiple MoAs to inhibit tumor growth. First, dual inhibition of both EGFR and cMet signaling by blocking ligand‐induced activation and inducing receptor degradation slows down tumor cell proliferation. Second, amivantamab has a low fucose N‐glycosylation in Fc, which enhances Fc and FcgR interactions on NK cells, monocytes, and macrophages to harness antibody‐dependent cellular cytotoxicity, antibody‐dependent cellular phagocytosis, and complement‐dependent cytotoxicity. In addition, Fc–FcgR interaction can lead to antibody‐dependent cellular trogocytosis, an antibody‐mediated transfer of membrane fragments and ligands from tumor cells to effector cells such as monocytes, macrophages, and neutrophils. The induction of trogocytosis can also lead to down‐regulation of EGFR and cMet receptors and their downstream signaling (Moores *et al*, 2016).

bsAbs could also bind to different epitopes of one target to block one pathway more efficiently through enhanced receptor internalization. Such biparatopic bsAbs targeting HER2 (zenocutuzumab, KN026) are now in clinical trials, and more such targets, such as VEGF, VEGFR2, and DLL4, are being investigated for similar approaches.

### Activating immune cells

T cells need to be carefully controlled during a defense action, and so‐called immune checkpoints are such regulators that switch T‐cell power on or off. Monoclonal antibodies against immune checkpoint regulators such as PD‐1, PD‐L1, or CTLA4 have been shown to display significant potencies in treating some cancers such as melanoma by activating T cells. However, they have limited effects in non‐inflamed or cold tumors, and resistance has emerged after mAb monotherapy. Thus, bsAbs targeting two immune checkpoints simultaneously may synergize their immune‐modulating functions. Moreover, monoclonal antibodies targeting co‐stimulatory receptors, such as OX40, ICOS, or CD28, may cause strong systemic side effects: The mAb TGN1412 for instance caused several deaths in clinical trials. Their toxicity might be limited locally by developing bsAbs that co‐target PD‐L1, the expression of which is restricted around the tumor microenvironment. So far only two checkpoint‐specific bsAbs are in phase 3 trials for treating solid tumors: KN046 (targeting PD‐L1/CTLA4) and tebotelimab (targeting PD‐1/LAG‐3).

## Clinical progress of bsAb

So far, there have been three bispecific antibodies approved by the FDA, two of them TRBA. Blincyto (Blinatumomab), bridging CD3 on T cells and CD19 on B cells, was first approved in 2014 for Philadelphia chromosome negative (Ph‐) relapsed or refractory B‐cell precursor acute lymphoblastic leukemia (ALL), based on a single‐arm phase 2 trial that showed 33% complete response rate (NCT02000427), and then expanded to Ph+ patients and patients in remission with minimal residual disease in 2017 and 2018, respectively. Removab (Catumaxomab), bridging CD3 and EpCAM, was approved for malignant ascites in 2009 and withdrawn after 4 years owing to commercial reasons, immunogenicity and toxicity. The third bsAb, an oncology‐unrelated product, Hemlibra (Emicizumab) was elegantly designed to prevent bleeding in hemophilia A patients and has been so far the most successful bsAb. It has changed the paradigm of hemophilia A treatment from three injections per week to once weekly or biweekly.

There are a few other promising bsAbs with different structures in phase 3 clinical trials including IgG‐like full‐length antibodies, DARTs, and fusion proteins. A lot more entities are being investigated in early phases now and may enter phase 3 investigation within the next 5 years. Some antibodies in early‐phase trials have been granted breakthrough therapy designation by FDA or enter other expedited programs (Table 1).

**Table 1:** Bispecific antibodies investigated in late‐phase clinical trials or granted expedited designations by FDA.

Antibodies	Targets	Structure and platform	Company	Indication	Pivotal trial(s)	Trial phase	FDA designation(s)
Glofitamab (Bacac *et al*, 2018; Hutchings *et al*, 2021; Killock, 2021)	CD3xCD20	Asymmetric (2 + 1)	Roche	DLBCL	NCT04408638	3	BTD
KN046 (Zhao *et al*, 2020)	PD‐L1xCTLA4	Asymmetric (1 + 1)	Alphamab	NSCLC	NCT04474119	3	··
··	··	··	··	Thymic carcinoma	NCT04469725	2	Orphan
IBI318 (Xu *et al*, 2020)	PD‐1xPD‐L1	Asymmetric (1 + 1)	Innovent	NSCLC	NCT04672928	1b/3	··
Epcoritamab (SC) (Engelberts *et al*, 2020; Hutchings *et al*, 2020; van der Horst *et al*, 2021)	CD3xCD20	Asymmetric (1 + 1, DuoBody)	Genmab	DLBCL	NCT04628494	3	··
Tebotelimab (Luke *et al*, 2020; Patel *et al*, 2020; Catenacci *et al*, 2021)	PD‐1xLAG‐3	Symmetric (2 + 2, DART)	MacroGenics	G/GEJ (+HER2Ab)	NCT04082364	2/3	··
··	··	··	··	G/GEJ (+PARPi)	NCT04178460	1	··
Tebentafusp (Damato *et al*, 2019; Middleton *et al*, 2020; Sacco *et al*, 2020)	TCR/CD3	Asymmetric (fusion protein)	Immunocore	Melanoma	NCT03070392	3	BTD
Faricimab (Khan *et al*, 2020; Khanani *et al*, 2020; Sahni *et al*, 2020)	Ang‐2xVEGF‐A	Asymmetric (1 + 1, CrossMab)	Roche	DME	NCT03622580 / NCT03622593	3	··
Amivantamab (Park *et al*, 2020; Vijayaraghavan *et al*, 2020; Yun *et al*, 2020)	EGFRxMETex20mut	Asymmetric (1 + 1, DuoBody)	Janssen	NSCLC	NCT02609776	3	BTD
Mosunetuzumab (Hosseini *et al*, 2020)	CD3xCD20	Asymmetric (1 + 1)	Roche	FL	NCT02500407	3	BTD
Zanidatamab (Meric‐Bernstam *et al*, 2021; Pant *et al*, 2021)	HER2 ECD2xECD4	Asymmetric (1 + 1)	Zymeworks	BTC	NCT04466891	2	BTD
Flotetuzumab (Uy *et al*, 2021)	CD3xCD123	Fragment (1 + 1, DART)	MacroGenics	AML	NCT02152956	1/2	Orphan
APVO436 (Godwin *et al*, 2017; Slade & Uy, 2020)	CD3xCD123	Symmetric (2 + 2)	Aptevo	AML	NCT03647800	1	Orphan
Zenocutuzumab (Cancer Discov, 2019; Pistilli *et al*, 2020; Schram *et al*, 2020)	HER2xHER3	Asymmetric (1 + 1)	Merus	Pancreatic cancer	NCT02912949	1/2	Orphan
··	··	··	··	NRG1 fusion cancers	NCT02912949	··	FT
TNB383B (Buelow *et al*, 2018; Rodriguez *et al*, 2020)	CD3xBCMA	Asymmetric (2 + 1)	AbbVie/TeneoBio	MM	NCT03933735	1	Orphan

AML, acute myeloid leukemia; BTC, biliary tract cancers; BTD, breakthrough therapy designation, FT, fast track; DLBCL, diffuse large B‐cell lymphoma; DME, diabetes macular edema; FDA, Food and Drug Administration; FL, follicular lymphoma; G/GEJ, gastric and gastroesophageal junction carcinoma; HER2Ab, HER2 antibody; MM, multiple myeloma; NSCLC, non‐small‐cell lung cancer; PARPi, poly‐ADP‐ribose polymerase inhibitor.

But there are also prominent setbacks. Roche’s Vanucizumab, an angiogenesis inhibitor targeting both Ang‐2 and VEGF‐A signaling, was terminated due to lack of progression‐free survival (PFS) improvement compared with bevacizumab in colorectal cancer patients. Faricimab, however, another Ang‐2/VEGF‐A blocker also developed by Roche, recently showed positive results in phase III studies for treating neovascular or “wet” age‐related macular degeneration (nAMD). The trials of duvortuxizumab and AFM11, both of which are closely similar to Blinatumomab, were terminated due to safety concerns. Lately, the programs of a few CD20XCD3 bispecific antibodies were either put on hold or terminated due to deaths in clinical trials, even though a high percentage of complete responses was observed. These examples show that, even with identical target combination and similar design, each bsAb may have unique properties owing to subtle differences. Careful optimization in molecule engineering and clinical dosing may rescue their applications, as Blincyto went through five phase I trials before moving further. M7824 (bintrafusp alfa; Strauss *et al*, 2018), composed of TGF‐βRII fused to a PD‐L1 antibody to target TGF‐β in tumor microenvironment (TME) and PD‐L1 for local immune modulation, unfortunately failed to improve overall survival (OS) and PFS in a phase 3 clinical trial (NCT03631706) in NSCLC compared with pembrolizumab. M7824 is still under investigation for biliary tract cancers and HPV‐related carcinomas.

## Designing the right T‐cell‐redirecting bsAbs

Currently all TRBAs are in phase I/II stage except for Blincyto. Similar to CAR‐T therapy, one of the biggest challenges and main safety concerns is the CRS. To address this problem requires designing an ideal bsAb molecule that is tunable in potency to generate optimal cytokine levels as extensively summarized recently (Middelburg *et al*, 2021).

There are a few relevant factors for designing a new generation of TRBAs. First is choosing the right CD3 binding arm. While CD3 seems to be the crucial target, the level of TRBA‐induced CRS correlates with the interaction mode and affinity of the anti‐CD3 antibody to CD3 subunits (Ɛ/γ or σ/γ). Indeed, strong anti‐CD3ɛ mAb (such as OKT3)‐derived TRBAs tend to cause severe CRS, as the T‐arm binds tightly to both CD3δɛ and CD3γɛ heterodimers. Exploring an alternative T‐arm, a recently developed anti‐CD3 clone (F2B) binds preferentially to CD3δɛ with intermediate affinity and has no detectable affinity to CD3γɛ heterodimer (Trinklein *et al*, 2019). Subsequently, F2B‐based TRBA reduced cytokines to minimal level and demonstrated satisfactory potency in animal models.

Second, the affinity gap between the TRBA to the TAA binding site and the anti‐CD3 unit should be at least 10‐fold to establish sequential binding so as to reduce the risk of a severe CRS (Mandikian *et al*, 2018). Individual TRBA molecule must bind cancer cell first; free TRBA should not be able to stably bind to T cells. TRBAs administered into the bloodstream would follow the flow and bind to target‐expressing cells. A cluster of bound TRBAs would then grab and enrich for cytotoxic T cells to be activated. This design reduces antigen‐independent activation and antibody‐induced cell death by the TRBA.

Lastly, the physical distance between the two antibody binding units is a double‐sided sword as it affects both potency and CRS induction (Ellerman, 2019). On the one hand, a short space between T‐arm and cancer cell‐arm allows efficient diffusion of the cytotoxic molecules (perforin and Granzyme B) into the malignant cell to achieve fast clearance of tumor mass. On the other hand, a short arm might induce a strong CRS. Finding the right distance between the two arms in TRBAs remains a delicate task.

Choosing the right targets in solid tumors can be even more challenging for TRBAs than classic mAbs, due to the induction of amplified T‐cell response *in vivo*. Candidate tumor antigens can be grouped as tumor‐specific antigen (TSA), tumor‐associated antigen (TAA), or cancer germline antigens (CGA), based on their expression patterns (Seremet *et al*, 2011; Abbott *et al*, 2020). TSAs, such as EGFRvIII (Gedeon *et al*, 2013), p95HER2 (Rius Ruiz *et al*, 2018), RAS G12V, or Q61H/L/R (Douglass *et al*, 2021), are ideal cancer antigens for TRBAs, since these are exclusively expressed on the tumor cell surface. However, very few TSA are available, and if there is one, antigen heterogeneity can be a problem (Furnari *et al*, 2015): *In vitro* experiments showed limited effects on brain metastases of p95HER2 TRBA (Rius Ruiz *et al*, 2018). More studies in both preclinical and clinical settings are needed to develop efficient TSA targeting antibodies.

The next ideal antigens are CGA with CD19 (Scheuermann & Racila, 1995) being the most frequently used in targeting B‐cell malignancies, but CGA are limited in non‐essential tissues. Tumor over‐expressed antigens or TAA, such as CEA and HER family members, can also be used for TRBA design. Additionally, proteins with aberrant post‐translational modifications such as MUC1 (Steentoft *et al*, 2019), intracellular oncoproteins such as WT1 (Dao *et al*, 2015), and tissue‐specific antigens such as PSMA, Mullerian inhibiting substance type II receptor (Bakkum‐Gamez *et al*, 2008) and others should be extensively explored. Generally, tumor antigens are expressed on the membrane of tumor cells; however, tumor antigens can also include post‐translationally modified products such as carbohydrate chains or even lipids. Meanwhile, new methods such as whole‐exome sequencing combined with mass spectrometry should be applied to discover novel antigens (Wang & Cao, 2020) in cancer cells for TRBA development.

Another design approach is Fc‐modification. All TRBAs currently under clinical investigations are devoid of the Fc or have mutations that attenuate their effector functions. This minimizes potential off‐target toxicity and prevents the unintended depletion of effector T cells. However, it makes “bridging” effector T cells and tumor cells the only MoA of these bsAbs, which may limit their therapeutic efficacy. It is therefore an interesting approach to design bsAbs that retain Fc‐mediated killing function specific against tumor cells but not T cells. Indeed, it has been found that the spatial distance between antigen epitope and effector T cells may be critical for effective initiation of T‐cell signaling (Srivastava & Riddell, 2015). Thus, it might be possible to design a bsAb with a unique structure that only allows Fc‐mediated effector activity against tumor cells captured by one arm of the bsAb but not against T cells bound to the other arm of the bsAb.

A perfect preclinical TRBA study does not necessarily guarantee its clinical success, particularly in solid tumors. TRBAs could only work through penetration into the core of a tumor mass to attract and activate T cells. Like other macro‐molecule therapies against solid tumors, the microenvironment poses a series of challenges for TRBA‐assisted T‐cell therapy. The physical barrier of a solid tumor hinders penetration as well as trafficking and infiltration of T cells, significantly reducing the number of both TRBA molecules and T cells inside. Moreover, the suppressive tumor microenvironment, including major cytokines such as TGF‐β (Caja *et al*, 2018), PD‐L1 (Jiang *et al*, 2019), and inhibitory cells such as Treg (Ohue & Nishikawa, 2019), all impair local and recruited T‐cell immune response.

Numerous approaches are now being investigated to tackle these challenges, including combining checkpoint inhibitors with TRBAs (Kobold *et al*, 2018); delivering BiTEs to solid tumors with new vehicles such as oncolytic viruses (Rosewell Shaw & Suzuki, 2018); supplementing the co‐stimulatory signals, for example, through CD28 signaling for complete T‐cell activation and combination therapy (Skokos *et al*, 2020); and more. Any clinical breakthrough that adopts one or more of these strategies will inform the further development of TRBAs to treat solid tumors.

## Co‐stimulatory TRBAs

While the main route to trigger T‐cell receptor (TCR; for instance via CD3) signaling is the specific recognition of cognate antigenic peptides presented by MHC molecules, co‐stimulatory / co‐inhibitory receptors on T cells direct T‐cell function and determine T‐cell fate besides the duration and amplitude of their activation / inhibition. This two‐signal mode of T‐cell activation has been well studied, and a plethora of stimulatory, co‐stimulatory, inhibitory, and co‐inhibitory receptors/ligands have been discovered (Chen & Flies, 2013). Among them, CD28 (Esensten *et al*, 2016) and CD137/4‐1‐BB (Long *et al*, 2015) and their ligands are the most extensively studied co‐stimulatory receptors for application in immunotherapy. The great value of co‐stimulation has been well validated since the successful development of 2nd‐generation CAR‐Ts, who must contain at least one co‐stimulatory domain in the activation cassette in addition to the CD3ζ domain.

The fact that CD3‐based TRBAs have demonstrated some success in clinical studies without co‐stimulatory signals actually questions the need for integrating additional components in anti‐tumor bsAbs. Yet, clinical safety concerns that halted the trials of MGD011 and AFM11 illustrate the daunting challenge of managing CRS‐related toxicity. Co‐stimulation, by its nature, may induce a milder T‐cell activation when compared to CD3‐based TRBAs such as BiTEs and DARTs. Co‐stimulating mAbs could also assist CD3‐TRBA activation—a similar role as in CAR‐Ts—if the latter is not fully mobilized to achieve satisfactory potency in treating solid cancer. For these reasons, co‐stimulation‐based TRBAs have attracted increasing attention from academia and industry in recent years.

There are three approaches to use co‐stimulation in order to improve the safety/efficacy of TRBAs: replacing, addition, and integration. Replacing means designing co‐stimulation/inhibitory‐based bsAbs instead of CD3‐based TRBAs. So far, CD28 (Waite *et al*, 2020), CD137 (Hinner *et al*, 2019), OX40 (Perez‐Santos, 2021), and PD‐L1 (Wang *et al*, 2021a) are frequently selected to replace CD3; other co‐receptors are being actively pursued. Other approaches use anti‐co‐stimulatory mAbs or co‐stimulatory‐TRBAs in combination with CD3‐TRBAs (Bohlen *et al*, 1997; Cochlovius *et al*, 2000; Claus *et al*, 2019). Integrating means designing multispecific antibodies, such as tri‐specifics that contain an anti‐co‐stimulatory binding moiety (Garfall & June, 2019; Mullard, 2020). While there are exciting results in animal experiments, most of these alternative TRBAs are still in preclinical stage with very few in early‐phase clinical trials.

## Management of the bsAb toxicity and resistance

Immunotherapeutic anti‐cancer agents, such as anti‐CD3 antibodies, CAR‐Ts, BiTEs, or non‐protein drugs, have an inherent risk of causing potentially fatal adverse effects, most notably CRS (Chatenoud *et al*, 1989; Shimabukuro‐Vornhagen *et al*, 2018). Interleukin‐6 (IL‐6), IL‐10, TNF‐alpha, and interferon (IFN)‐gamma are among the central cytokines that are consistently found elevated in the serum of CRS patients. Some patients also suffered from neurotoxical side effects after administration of BiTEs and TRBAs.

Severe CRS is a life‐threatening situation that requires prompt and aggressive treatment. Clinical CRS management is still evolving as CAR‐Ts, BiTEs, TRBAs, and other agents advance in clinical trials. As most CAR‐T‐treated patients have elevated serum IL‐6, anti‐IL‐6 (Siltuximab) and anti‐IL‐6R antibody (Tocilizumab) have been used to manage severe CRS (Riegler *et al*, 2019). When neither tocilizumab nor glucocorticoids were effective, TNF‐α signaling blockade has also demonstrated effectiveness. It is unknown whether antibodies to other cytokines are helpful to manage CRS. For Blinatumomab, a prophylactic protocol consisting of cytoreduction, dose adjustment, and premedication with corticosteroids has been suggested based on the clinical trials (Topp *et al*, 2015). This experience should serve as a guideline to manage CRS in other TRBA‐treated patients. The CRS severity is likely to increase with combination therapy. Odronextamab (REGN1979), a TRBA antibody redirecting T cells to CD20, demonstrated an increased level of CRS‐related toxicity in patients when combined with a PD‐1 antibody, which caused two deaths in trials. This is once again a warning of the daunting task of managing clinical toxicity of TRBAs.

Emerging resistance to bsAbs has also limited their clinical applications. Loss of target, inadequate immune response, and upregulation of immune checkpoints or other immune escape‐related pathways were among the mechanisms observed. Extramedullary escape in CNS and testes was a common resistance mechanism in treating hematological malignancies with bsAbs. Poor infiltration of redirected lymphocytes, and immunosuppressive tumor microenvironment were common problems in solid tumors.

## Perspectives

With hundreds of TRBAs in clinical trials, there is great hope that some of them may lead to breakthroughs in cancer therapy. Particularly, the long‐lasting IgG‐based bsAbs are promising as the drug exposure time extends significantly *in vivo*, allowing durable interactions between immune cells and target cells, or effective dual regulation of two vital signals.

Another advance that extends the cell‐bridging effect is smart delivery of TRBAs. Instead of infusion as soluble protein, bsAb can be delivered through cell‐expressing vehicles. In this regard, one could consider T‐cell receptor (TCR) engineered cells, CAR‐Ts, and cell‐infecting viruses as living TRBAs (Chen *et al* 2021; Wang *et al*, 2021b). One study demonstrated that EGFR‐targeted BiTEs produced by CAR‐T cells could induce effective and specific anti‐tumor activity against heterogeneous tumors and mitigate against the effects of EGFRvIII which causes antigen loss. Local BiTE secretion could enable targeting of an antigen, such as EGFR, that would otherwise not be “druggable” by BiTEs or CAR‐Ts due to the off‐target toxicity (Choi *et al*, 2019). Additionally, these vehicles may have advantages for targeting multiple antigens. A study found that T cells co‐expressing IL‐13Rα2‐CARs and HER2 showed improved tumor control in a human glioblastoma multiforme (GBM) orthotopic xenograft model (Hegde *et al*, 2013). Indeed, delivering BiTEs or TRBAs with new vehicles, as well as supplementing T cells with co‐stimulatory signals beside CD3‐based activation, could become promising therapeutic approaches.

Meanwhile, it is well understood that no single drug can win the war against cancer. Combination of multiple remedies is the current trend in cancer treatment, but the combination strategy should be carefully designed, and must fundamentally be science‐based. In principle, drugs with independent MoAs can work together to generate synergistic effects. TRBAs might combine with an anti‐PD‐L1 antibody to achieve better outcome (Gedeon *et al*, 2013); a MoA that delivers mutual enhancement is likely to achieve synergy. Combination treatment of anti‐PD‐1 antibody with Lenvatinib already demonstrated enhanced anti‐cancer effect in a tumor model (Kato *et al*, 2019), as the latter selectively inhibits VEGFR1–3 and other proangiogenic and oncogenic pathway‐related receptor tyrosine kinases (RTKs), thus facilitating the traffic of immune T cells into the tumor microenvironment.

As antibody technology develops, several trispecific or even multispecific antibodies are being investigated in clinical trials, including both full‐length and fragment‐based platforms. Two main strategies were adopted, either combining more than one immune cell target such as SAR442257 (CD38×CD28×CD3; El‐Murr *et al*, 2020) and GTB‐3550 (CD16xCD33xIL‐15) to recruit immune cells more efficiently, or adding on HSA (Human Serum Albumin) to enhance the bioavailability, such as ND‐021 (PD‐L1x4‐1BBxHSA; Sahni *et al*, 2020) and HPN‐328 (DLL3xAlbxCD3). Inspired by efforts from the fight against HIV, many trispecific antibodies are now under investigation for treating malignant tumors.

## Conflict of interest

The authors declare that they have no conflict of interest.

## For more information

i www.fda.gov
ii cde.org.cn
iii clinicaltrials.gov

**Pending issues**

- One of the biggest challenges and main safety concerns is the CRS. Addressing this issue will require designing bsAb molecule tunable in potency to generate optimal cytokine levels.
- Choosing the right target in solid tumors can be even more challenging for TRBAs than for classic mAbs, due to the induction of amplified T‐cell response *in vivo*, and will require particular attention.
- TRBAs need to penetrate into the core of a tumor mass to attract and activate T cells. The microenvironment thus poses a series of challenges for TRBA‐assisted T‐cell therapy that need to be overcome.
